# Subcellular Location of *Piscirickettsia salmonis* Heat Shock Protein 60 (Hsp60) Chaperone by Using Immunogold Labeling and Proteomic Analysis

**DOI:** 10.3390/microorganisms8010117

**Published:** 2020-01-15

**Authors:** Cristian Oliver, Patricio Sánchez, Karla Valenzuela, Mauricio Hernández, Juan Pablo Pontigo, Maria C. Rauch, Rafael A. Garduño, Ruben Avendaño-Herrera, Alejandro J. Yáñez

**Affiliations:** 1 Laboratorio de Inmunología y Estrés de Organismos Acuáticos, Instituto de Patología Animal, Facultad de Ciencias Veterinarias, Universidad Austral de Chile, Valdivia 5090000, Chile; cristianoliver7@gmail.com; 2 Interdisciplinary Center for Aquaculture Research, (INCAR), Concepción 4070386, Chile; patricio.sanchez@uach.cl; 3 Instituto de Bioquímica y Microbiología, Facultad de Ciencias, Universidad Austral de Chile, Valdivia 5090000, Chile; jppontigo@gmail.com (J.P.P.); crauch@uach.cl (M.C.R.); 4 Microbiology and Immunology Department, Dalhousie University, Halifax, NS B3H 4R2, Canada; karla.valenzuela.tm@gmail.com (K.V.); rafael.garduno@dal.ca (R.A.G.); 5 Austral-OMICS, Faculty of Sciences, Universidad Austral de Chile, Valdivia 5090000, Chile; mauricio.hernandez@uach.cl; 6 Canadian Food Inspection Agency, Dartmouth Laboratory, Dartmouth, NS B3B 1Y9, Canada; 7 Universidad Andrés Bello, Laboratorio de Patología de Organismos Acuáticos y Biotecnología Acuícola, Facultad Ciencias de la Vida, Viña del Mar 2531015, Chile; 8 Facultad de Ciencias, Universidad Austral de Chile, Valdivia 5090000, Chile

**Keywords:** *Piscirickettsia salmonis*, SRS, fish pathology, IgY, vaccination, secretion systems

## Abstract

*Piscirickettsia salmonis* is the causative bacterial agent of piscirickettsiosis, a systemic fish disease that significantly impacts the Chilean salmon industry. This bacterium possesses a type IV secretion system (T4SS), several proteins of the type III secretion system (T3SS), and a single heat shock protein 60 (Hsp60/GroEL). It has been suggested that due to its high antigenicity, the *P. salmonis* Hsp60 could be surface-exposed, translocated across the membrane, and (or) secreted into the extracellular matrix. This study tests the hypothesis that *P. salmonis* Hsp60 could be located on the bacterial surface. Immunogold electron microscopy and proteomic analyses suggested that although *P. salmonis* Hsp60 was predominantly associated with the bacterial cell cytoplasm, Hsp60-positive spots also exist on the bacterial cell envelope. IgY antibodies against *P. salmonis* Hsp60 protected SHK-1 cells against infection. Several bioinformatics approaches were used to assess Hsp60 translocation by the T4SS, T3SS, and T6SS, with negative results. These data support the hypothesis that small amounts of Hsp60 must reach the bacterial cell surface in a manner probably not mediated by currently characterized secretion systems, and that they remain biologically active during *P. salmonis* infection, possibly mediating adherence and (or) invasion.

## 1. Introduction

Piscirickettsiosis is caused by the facultative intracellular Gram-negative bacterium *Piscirickettsia salmonis* [1,2,3]. *P. salmonis* is currently the primary bacterial pathogen affecting farmed salmonids (*Oncorhynchus kisutch*, *Salmo salar*, and *Oncorhynchus mykiss*) in southern Chile [4]. Piscirickettsiosis is characterized by septicemia of the kidney, liver, spleen, intestine, brain, ovary, and gills [5]. Antibiotics and vaccination are commercially used to treat and prevent this disease, albeit with limited success [6]. Since antibiotic treatments can lead to drug residues in animal products and result in antibiotic resistance of the pathogen [7,8,9], the formulation of improved and more effective vaccines is desirable.

Although numerous virulence-related genes have been identified in *P. salmonis* [10,11], few of the encoded virulence factors have been characterized. Particularly relevant are evolutionarily conserved molecular chaperones of the heat shock protein (HSP) family, which modulate protein folding, multimeric protein assembly/disassembly, protein translocation across membranes, protein degradation, and signal transduction [12]. Several HSPs are also moonlighting proteins that can exhibit more and novel biological functions, thus extending the range of the functional proteome [13].

The bacterial 60-kDa HSP (Hsp60, also known as GroEL), a highly conserved protein and dominant antigen of most pathogenic bacteria, is involved in the pathogenesis of several infectious diseases. Furthermore, surface-associated Hsp60 is involved in host-cell adhesion and invasion [14,15], as well as in modulating the host immune response [16]. Hsp60 is secreted into the extracellular space or pathogen-containing host-cell vacuoles during infection by *Legionella pneumophila* [14], and *Helicobacter pylori* [17], among others. Interestingly, *L. pneumophila* Hsp60 can recruit mitochondria to the vacuole and remodel the actin cytoskeleton in infected Chinese hamster ovary cell lines [18], most likely by interacting with the host proteins [19].

*P. salmonis* Hsp60 is highly immunogenic [20], and recombinant Hsp60 raises an antibody response in Atlantic salmon [21]. Indeed, a vaccine based on a mixture of the recombinant *P. salmonis* Hsp60 and Hsp70, as well as the flagellar protein FlgG, elicits a strong protective humoral response in challenged fish [22]. Besides their potential vaccination benefits, the high antigenicity of *P. salmonis* Hsp60 suggests exposure on the bacterial cell surface. However, the subcellular location and secretion mechanisms of *P. salmonis* Hsp60 have not been examined. This study was designed to test the hypothesis that Hsp60 is a putative virulence effector protein secreted by *P. salmonis,* hence, its value as a vaccine target must be considered.

## 2. Materials and Methods

### 2.1. Bacterial Strains and Cell Line

The *P. salmonis* LF-89^T^ (ATCC VR-1361) type strain was routinely grown in AUSTRAL-SRS broth at 18 °C for five days [3]. The *P. salmonis* AUSTRAL-005, AUSTRAL-006, and AUSTRAL-010 strains, isolated from Chilean salmon farms, were used for western blot analysis, and AUSTRAL-005 was used for inhibitory efficacy experiments. The strains’ identities were confirmed by biochemical procedures, PCR assays, and 16S rRNA sequencing [23]. The SHK-1 cell line (ECACC 97111106, 40-50 passages), derived from Atlantic salmon embryos, was used as a model for in vitro infection. SHK-1 cells were grown in Leibovitz’s L-15 (Gibco BRL) supplemented with 10% fetal bovine serum (HyClone) at 18 °C in aerobic conditions [24].

### 2.2. Ethics Statement

All experimental protocols complied with guidelines for the use of laboratory animals, as established by the Chilean National Commission for Scientific and Technological Research (CONICYT, Spanish acronym) and the Universidad Austral de Chile Bioethics Sub-Committee and were fully approved by this institution for the present project (FONDAP-INCAR 15110027, renewed in November 2018).

### 2.3. In Silico P. salmonis Hsp60 Amino Acid Sequence Analyses

Multiple sequence alignments were performed with the Clustal Omega tool [25] (v1.2.1). Protein sequence identity and similarity calculations were carried out using the MatGAT v2.0.2 tool [26]. The Hsp60 sequences from several bacteria (Genbank Acc. AAV80377—*P. salmonis*; CAA67358—*Francisella tularensis*; KER64311—*Aeromonas hydrophila*; KFN17454—*Aeromonas salmonicida*; AAG48876—*Vibrio vulnificus*; ACA58291—*Vibrio anguillarum*; KKM58231—*Yersinia pestis*; AAL55999—*Escherichia coli*; AEV64537—*Pseudomonas fluorescens*; AAB34346—*Pseudomonas aeruginosa*; AQL11108—*Legionella pneumophila*; ATN80743—*Coxiella burnetii*; AIH51208—*Mycobacterium tuberculosis*; ANH47662—*Helicobacter pylori*) were aligned with the Clustal Omega tool [25] (v1.2.1) and trimmed manually by deleting gaps in the alignments. The set of sequences was then analyzed using PhyML v3 [27] with a LG+I+G+F (Le-Gascuel + invariant + gamma + frequency-empirical) model and 1000 bootstrap replications. Bayesian inference analysis was computed using MrBayes software (v3.2.6) [28] under a LG+I+G+F substitution model for 10^5^ generations, burn-in 25%. Amino acid sequences were also analyzed with the T3S effector prediction programs SIEVE [29], pEffect [30], EffectiveT3 [31] and ModLab T3S [32], as well as the T4S effector prediction programs T4SEpre [33] and T4 EffPred [31], and the T6SS predictor tools Bastion6 [34] and SecReT6 [35]. Amino acid sequences for *L. pneumophila* VipE, *Salmonella enterica* SopE and *Legionella londiniensis* Hcp were used as positive controls for the T3SS, T4SS, and T6SS effector predictors, respectively.

### 2.4. Immunogold Labelling of P. salmonis Hsp60

In vitro-grown bacteria were fixed in freshly depolymerized 4% (*w*/*v*) paraformaldehyde and 0.5% (*v*/*v*) glutaraldehyde in 0.1 M sodium cacodylate buffer (pH 7.2), post-fixed in 0.25% (*w*/*v*) aqueous uranyl acetate to stabilize phospholipids and enhance membrane contrast, and, finally, embedded in either epoxy resin (TAAB 812 resin, Marivac Ltd.) or LR white resin. Ultrathin sections were picked up on 200-mesh nickel grids. Immunolabeling was performed at room temperature (RT) [14]. Briefly, the grids were floated on PBS containing 1% (*w*/*v*) bovine serum albumin (BSA) in 24-well plates and blocked for 1 h. Grids were then incubated overnight at 4 °C with mild agitation, followed by 2 h at 37 °C on 300 µL drops of a monoclonal anti-Chlamydia trachomatis Hsp60 antibody (Abcam) (1:300 in PBS containing 0.1% (*w*/*v*) BSA). Grids were then floated for 1 h on drops of an anti-mouse IgG antibody conjugated to 10-nm colloidal gold spheres (1:100 in PBS containing 0.1% (*w*/*v*) BSA) (Sigma Immunochemicals, St. Louis, MO, US). Unbound antibodies were washed off by floating grids on 1 mL PBS-0.1% BSA. Specimens were then fixed by floating the grids on drops of 2.5% (*v*/*v*) glutaraldehyde in PBS for 10 min. Grids were repeatedly washed on drops of deionized water, and then stained with 2% (*w*/*v*) aqueous uranyl acetate and a modified Sato’s lead stain [36] before observation in a JEOL JEM-1320 transmission electron microscope (80 kV), and image capturing using a Hamamatsu ORCA-HR digital camera. Control experiments included bacteria labeling with gold conjugates in the absence of the primary antibody or labeling with irrelevant antibodies (rabbit anti-FlgG serum, 1:400 in PBS-0.1% BSA).

**Figure 1:**
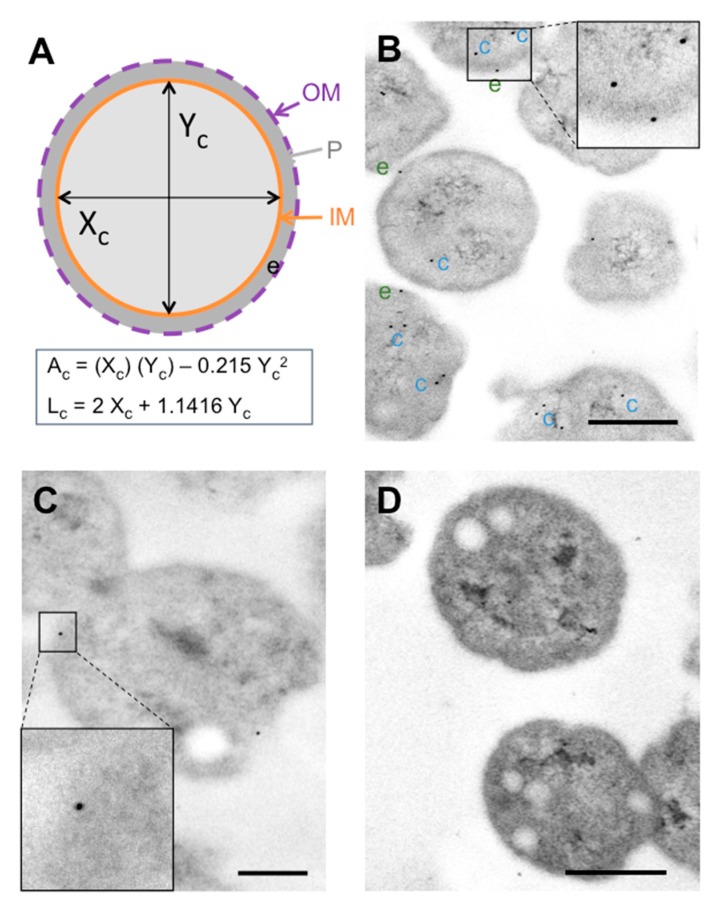
Subcellular localization of *P. salmonis* Hsp60 by immunogold analysis. (**A**) Schematic representation of a bacterial cell section indicating the two measurements taken (X_c_, Y_c_). The formulas used to calculate the areas of the cytoplasmic and periplasmic compartments are indicated below the scheme, where Ac is the cytoplasm area; Lc is the cytoplasmic membrane length, and e is the envelope. (**B**) Representative electron micrograph showing labeling patterns of ultrathin section cuts from *P. salmonis* LF-89^T^. Hsp60-positive spots are indicated with “c” (cytoplasm, blue) and “e” (envelope, green). Inset: magnification of Hsp60-positive spots. Bar scale = 0.5 μm. (**C**) Ultrathin section cuts from *P. salmonis* LF-89^T^ positive to FlgG, used as a control of cell envelope labeling. Inset: representative image of positive spot in cell envelope. Bar scale = 0.25 μm. (**D**) Representative background labeling of ultrathin *P. salmonis* LF-89^T^ section. Bar scale = 0.5 μm.

To facilitate relative labeling-pattern comparisons, subcellular gold particle distributions were standardized to dimensions of a “typical” bacterial section, calculated for each *P. salmonis* strain. Two measurements were taken in each bacterial cell section (Figure 1A), and the number of gold particles quantified in those sections was assigned to one of the following cellular compartments: cytoplasm, cell envelope (comprising the inner/outer membrane and periplasm), and extracellular surface. Gold particles not touching the inner membrane (IM) from the cytoplasm side were counted as part of the cytoplasmic compartment, whereas those touching the cytoplasmic membrane from either the cytoplasm or periplasm were counted as pertaining to the cell envelope. Gold particles touching the outer membrane from the periplasm were counted as belonging to the cell envelope. Particles on or touching (from the outside) the outer membrane were counted as part of the extracellular space (surface exposed). Given that primary and secondary antibody-gold conjugates may span some 20 nm, particles located on the extracellular surface that were not in contact with the outer membrane, but at a distance of ≤2 gold particle diameters, were still counted as part of the cell envelope.

### 2.5. Subcellular Fractionation and Protein Extraction for Proteomic Analyses

Subcellular fractionation was performed following the procedure reported [37]. Briefly, 100 mL of bacterial cells were pelleted at 5000× *g* for 10 min at 4 °C. Cells were resuspended in 500 µL of 0.2 M Tris-HCl (pH 8.0), 1 M sucrose, 1 mM EDTA. Then, 100 µL of lysozyme (5 mg/mL in deionized water) was added, the solution was vortexed and incubated for 5 min at RT. After 2 mL of deionized water was added, the solution was incubated at RT until spheroplasts were seen under the light microscope. Spheroplasts were pelleted by centrifugation at 5000× *g* for 15 min. The pellet was used to obtain cytoplasmic proteins and proteins associated with the IM. The supernatant (containing periplasmic proteins and outer membrane fractions) was ultracentrifuged (85,000× *g*, 30 min, 4 °C) to separate soluble and particulate material. The ultracentrifugation supernatant (periplasmic proteins) was stored at −20 °C until use. The ultracentrifugation pellet (containing OM proteins) was washed in 750 µL of washing solution (50 mM Tris-HCl (pH 8.0), 2% (*w*/*v*) Triton X-100, 10 mM MgCl_2_), pelleted by ultracentrifugation (85,000× *g*, 20 min, 4 °C) and stored at −20 °C until use.

Before processing for protein analysis, fractions were treated as follows: Washing solution (3 mL), and 50 µL DNase I (1 mg/mL in dH_2_O) were added to the OM pellet and mixed until suspension became clear. Then, the mixture was ultracentrifuged (85,000× *g*, 30 min, 4 °C). The supernatant was removed and stored at −20 °C until use. The pellet containing the IM was washed in 750 µL washing solution, and re-centrifuged at 85,000× *g* for 20 min at 4 °C. The supernatant of cytoplasmic proteins was removed and stored at −20 °C. The pellet containing the IM was then resuspended in 0.1 M sodium carbonate (pH 11.0) to a final volume of 60 mL and stirred for 1 h at 4 °C. Then, the suspension was ultracentrifuged at 120,000× *g* for 1 h at 4 °C. The pellet (IM proteins) was washed in 2 mL 0.1 M Tris-HCl (pH 7.3), centrifuged at 85,000× *g* for 20 min at 4 °C, and was finally washed three times in 500 µL deionized water and stored at −20 °C until use.

### 2.6. Lysis of Protein Samples for Mass Spectrometry

Protein samples obtained from different subcellular compartments were lyophilized and resuspended in 6 M guanidine hydrochloride, 25 mM NH_4_HCO_3_ (pH 7.5), reduced with 2 mM dithiotreitol (DTT) for 30 min at RT, and then alkylated with 10 mM iodoacetamide for 30 min in darkness at RT. The reaction was diluted seven-fold with 25 mM NH_4_HCO_3_ (pH 7.5), and 2 µL of 0.1 ng/mL of modified trypsin (Promega, Madison, WI, US) was added. The trypsin reaction was incubated for 16 h at 37 °C. The reaction was terminated by the addition of acetic acid (pH 2.0).

### 2.7. Protein Identification and Analysis

After the lysis, samples were concentrated on a Centrivap concentrator (Labconco, Kansas City, MO, US) to a final volume of 20 µL and loaded on a 350 µm inner diameter fused silica 2D HPLC triphasic peptide trap column packed in-house with 3 cm of a desalting C18 reversed-phase (100Å, 5 µm, Magic C18 particles, Michrom Bioresources, Auburn, CA, US), followed by 3 cm of a strong cation exchange column (300Å, 5 µm PolySULFOETHYL A, PolyLC Inc., Columbia, MD, US) and finally with 3 cm of resolving C18 reversed-phase. The peptide trap was mounted on the loop of a nanoLC (ThermoFinnigan, San Jose, CA, US). Following a wash with 0.1% formic acid for 30 min at 0.5 µL/min, the efflux of the peptide trap column was directed to a 10 cm resolving reversed-phase column (100Å, 5 µm, Magic C18 particles, Michrom Bioresources), mounted on the electrospray stage of an LTQ Velos PRO mass spectrometer (ThermoScientific, Waltham, MA, US).

The peptides were separated by a 0–90% acetonitrile gradient in 280 min at a flow rate of 350 nL/min. An electrospray voltage of 1.9 kV was used, with the ion transfer temperature set to 250 °C. The mass spectrometer was controlled by the Xcalibur software to perform continuously mass scan analysis on the Fourier transform (FT) followed by MSMS scans on the ion trap of the twelve most intense ions, with a dynamic exclusion of one repeat scans of the same ion, 30 s repeat duration, and 90 s exclusion duration. Normalized collision energy for MS/MS was set to 35%.

### 2.8. Data Analysis

All tandem mass spectra MS/MS samples were analyzed using Sequest (v2.1; Thermo Fisher Scientific). Sequest was set up to search uniprotPiscirickettsia+salmonis.fasta (10,012 entries) assuming the digestion enzyme trypsin. Sequest was searched with a fragment ion mass tolerance of 0.80 Da and a parent ion tolerance of 50 PPM. Carbamidomethyl of cysteine, deamidation of asparagine and glutamine, and oxidation of methionine were specified in Sequest as variable modifications.

### 2.9. Generation of IgY Against P. salmonis Hsp60

A total amount of 250 µg of a synthetic peptide corresponding to residues 103 to 131 of the Hsp60 N-terminal region (GVKSVAAGMNPMDLKRGIDKATIAAVAAL) was emulsified in 500 µL of Titermax (Sigma, St. Louis, MO, USA) and injected into the pectoral muscle of a 21-week-old laying white leghorn hen (Avian Pathology Laboratory, Institute of Animal Pathology, Universidad Austral de Chile). After this primary immunization, two equal booster inoculations were given after 3 and 7 weeks. Eggs from immunized and non-immunized (control) hens were collected and pooled at 1-week intervals after the primary immunization. Crude antibody from the yolk was extracted using the water-soluble fraction protocol [38], with some modifications. Briefly, yolks were separated from the egg white, diluted at 1:9 ratios with distilled water, and incubated overnight at 4 °C. The IgY-containing liquid phase was filtered (0.22 µm membrane). After centrifugation (10,000× *g*, 30 min, 4 °C) to eliminate contaminating particulate material, the water-soluble IgY fraction was collected, and further precipitated using a 33% (*v*/*v*) saturated ammonium sulfate solution. The pellet was resuspended with 65% (*v*/*v*) saturated ammonium sulfate and centrifuged (10,000× *g*, 30 min, 4 °C). Finally, the IgY-containing pellet was resuspended in an antibody buffer, dialyzed against PBS at 4 °C, and stored at −20 °C before use. The IgY against *P. salmonis* Hsp60 was evaluated by western blot against total proteins from strains AUSTRAL-005, AUSTRAL-006, and AUSTRAL-010.

### 2.10. Antigenicity Analyses of P. salmonis Hsp60 Synthetic Peptide by qRT-PCR

SHK-1 cells (1 × 10^5^/well) were seeded onto six-well plates and cultured in L-15 supplemented with 10% fetal bovine serum for three to four days until reaching ~90% confluence. Cell monolayers were incubated with the *P. salmonis* Hsp60 synthetic peptide (100 µM) for 72 h at 18 °C in the presence/absence of the NF-kB inhibitor BAY 11-7082 (1 μM; Sigma-Aldrich). Then, cells were rinsed with PBS (0.01 M; pH 7.4) and processed for total RNA extraction.

RNA was extracted from (i) stimulated, (ii) stimulated and inhibitor-treated, and (iii) non-stimulated (control) cells using the TRIzol Reagent (1000 µL; Ambion, Thermo Fisher Scientific, Waltham, MA, US). The extract was mixed with a chloroform solution (200 µL), vortexed for 15 s, and centrifuged at 12,000× *g* for 20 min at 4 °C. The supernatant was recovered, mixed with 2-propanol (500 µL), and incubated overnight at −20 °C. Total RNA was extracted using the AxyPrep Multisource RNA Kit (Axygen). To ensure no DNA contamination, total RNA (5 µg) from all samples was incubated at 37 °C for 10 min with DNase (Epicenter) in a final volume of 50 µL. To obtain cDNA, total RNA (1 µg) from each sample was reverse transcribed using M-MLV Reverse Transcriptase (Promega) following manufacturer instructions. Briefly, each RNA sample (1 µg) was combined with a 1 nM mix of random primers and oligo(dT) (from 15-27 bp) and incubated at 70 °C for 5 min. Then, a secondary solution containing 5 µL reverse transcriptase 5X reaction buffer, 1.25 µL 10 mM dNTPs, and 0.75 µL 40 U/µL RNaseOUT (Invitrogen) was added, adjusted with nuclease-free water to a final volume of 25 µL, and incubated for 60 min at 37 °C.

qRT-PCR analyses of each cDNA (1 µL) sample were performed as follows: initial denaturation at 95 °C for 5 min, denaturation at 95 °C for 15 s, annealing at 60 °C for 15 s, extension at 72 °C for 15 s, and a final extension at 72 °C for 5 min after 40 cycles. The SYBR Green Kit (2X) (Axygen) was used for qRT-PCR analyses, and the melting curve (55–95 °C) was determined to evaluate primer specificities, using 0.5 µM of each primer (see Supplemental Table S1). Target genes were normalized against two endogenous housekeeping genes, 23S ribosomal RNA and ß-actin. Three independent experiments were performed in triplicate. The calculated results are expressed as relative fold-changes [39].

### 2.11. Inhibitory Activity of IgY Anti-Hsp60 in SHK-1 Cells

IgY anti-Hsp60 inhibitory activity was assessed [24]. Briefly, SHK-1 cells (1 × 10^5^/well) were seeded onto poly-L-lysine-coated coverslips on 24-well plates containing L-15 supplemented with 2% fetal bovine serum and then cultivated to 80–90% confluence. For assays, monolayers of adherent SHK-1 cells were pre-incubated for 1 h with IgY against *P. salmonis* Hsp60 (1 µg/µL) and then infected for 3 h with exponential growth-phase *P. salmonis* AUSTRAL-005 (MOI 20). After this period, SHK-1 cells were washed twice with PBS, and the culture medium was replaced with L-15 containing 2% heat-inactivated fetal bovine serum. Controls included (i) cells treated with IgY purified from eggs of non-immunized hens; (ii) cells treated with an irrelevant IgY against fructose-1,6-bisphosphatase 1; (iii) cells without IgY treatment; and (iv) non-infected cells. Cell morphology was evaluated by inverted microscopy for up to eight days post-infection. Additionally, levels of the cytosolic enzyme LDH released into the supernatant were determined using the LDH cytotoxicity detection kit (Takara Bio Inc.) following manufacturer recommendations. Cytotoxicity (%) was calculated by simultaneously running a 100% LDH control of SHK-1 cells lysed with Triton X-100.

### 2.12. Statistical Analyses

Unless otherwise stated, three independent experiments were routinely performed in duplicate. Post-*P. salmonis* infection values were compared against the untreated control and analyzed using a Student’s t-test. *p*-values ≤ 0.05 were considered statistically significant.

## 3. Results

### 3.1. Labelling Patterns of In Vitro-Grown P. salmonis

Polyclonal antibody labeling showed Hsp60 epitope predominance in association with *P. salmonis* cytoplasm (Figure 1B), counting gold particles not touching the inner membrane (IM) from the cytoplasm. Hsp60-positive spots were identified in the cell envelope, but only a few were found on the extracellular surface. Positive spots were also identified on *P. salmonis* outer membrane vesicles (OMVs) (Supplementary Figure S2). The Hsp60 labeling pattern was confirmed by those obtained with rabbit antisera against FlgG (Figure 1C). Standardization indicated that most of the antibody-recognized epitopes (~60%) were in cytoplasmic locations (Table 1), while ~28% were identified in the cell envelope, and ~11% were located on the extracellular surface. The results were standardized to the dimensions of the typical *P. salmonis* section (averaged from three labeling experiments comprising a total of 180 bacterial cell sections), with a cellular area of 0.5 ± 1.79 µm^2^, and a cellular membrane length of 2.56 ± 0.45 µm^2^ (Figure 1A). Background (non-specific) labeling was restricted to the cytoplasmic area (Figure 1D), and the chance of finding a random gold particle in relation to the cell envelope was estimated at <1.5%. Considering that control labeling was run for every condition, the background labeling was subtracted from specific monoclonal antibody (mAb) labeling to obtain the values presented in Table 1. Unexpectedly, no increase was found for bacterial Hsp60 within the cell (data not shown). Additionally, proteomic analyses identified 62 peptides corresponding to Hsp60 in different subcellular locations of *P. salmonis* (Supplementary Table S2).

### 3.2. Subcellular Fractionation and Proteomic Analyses

The mass spectrometry analysis of each *P. salmonis* compartment shows that Hsp60 is present in all subcellular locations. Hsp60 was found predominantly in the cytoplasm, but peptides were also detected in regions such as the periplasm, inner membrane and, interestingly, in the cell envelope (Table 2). Moreover, high amounts of Hsp60 peptides were detected in the OMVs from *P. salmonis* when compared to cellular compartments other than the cytoplasm. This confirms the presence of *P. salmonis* Hsp60 in extra cytoplasmic locations, corroborates the labeling patterns, and therefore suggests that Hsp60 is exported in *P. salmonis* via a yet unknown mechanism.

### 3.3. In Silico Analyses of P. salmonis Hsp60 Amino Acidic Sequence

**Figure 2:**
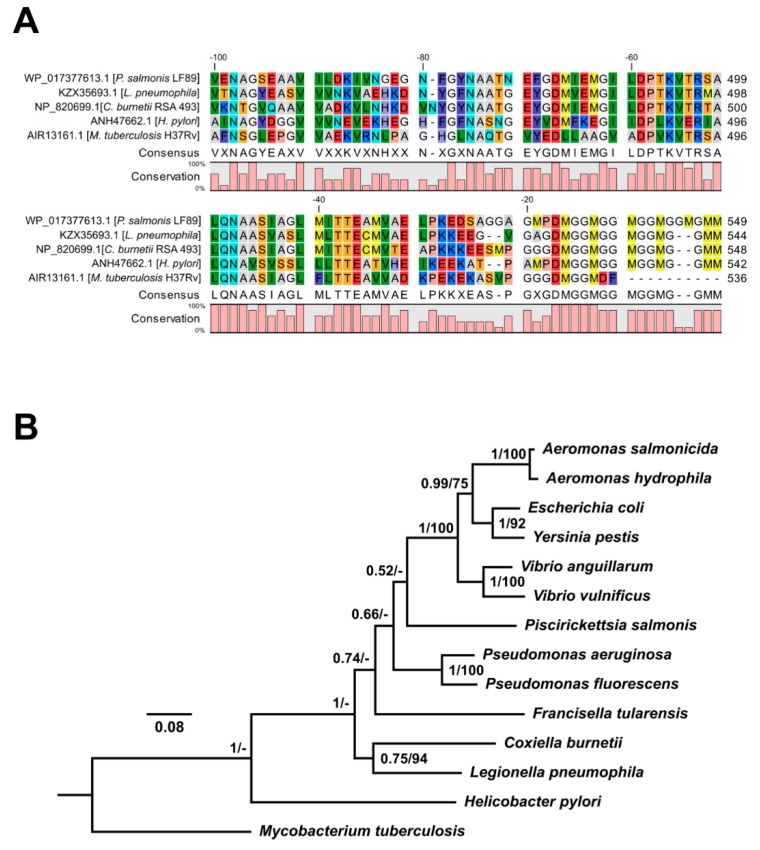
Amino acid sequence analysis of C-terminal region of Hsp60 from *P. salmonis*. (**A**) Multi-alignment of 100 amino acid residues at the C-terminus of Hsp60 for *P. salmonis*, *L. pneumophila*, *C. burnetii*, *H. pylori*, and *M. tuberculosis* using the ClustalOmega tool. Residue colors correspond to the RasMol scheme. (**B**) Phylogenetic analysis of *P. salmonis* Hsp60 sequence and homologs from several related bacteria. The consensus tree was constructed using the Bayesian inference method. Bayesian posterior probabilities (>0.5) and maximum likelihood bootstrap support values (>50%) are indicated at each node.

The identity matrix (Table S3) revealed high identity (57–74%) and similarity (73.4–86.3%) levels among bacterial Hsp60 sequences from *P. salmonis*, *C. burnetii*, *L. pneumophila*, *H. pylori*, and *M. tuberculosis*, even between distant species. Likewise, multiple alignment of the C-terminal region of related Hsp60 sequences supported a high degree of conservation (Figure 2A). Phylogenetic analyses placed *P. salmonis* Hsp60 in a clade with Hsp60 homologs present in other gamma proteobacteria, supported by strong bootstrap values (Figure 2B). To better assess the T3SS or T4SS effector potential of Hsp60 from *P. salmonis* and related bacteria, various bioinformatics prediction tools were used. Results were negative for all Hsp60 sequences analyzed for T3SS, T4SS, and T6SS (Supplementary Tables S4–S6 in Supplementary Materials) predictions. These data suggest that *P. salmonis* Hsp60 is not an effector translocated by the aforementioned secretion systems but employs a different mechanism for translocation.

### 3.4. Inhibitory Activity of IgY Anti-Hsp60 From P. salmonis

**Figure 3:**
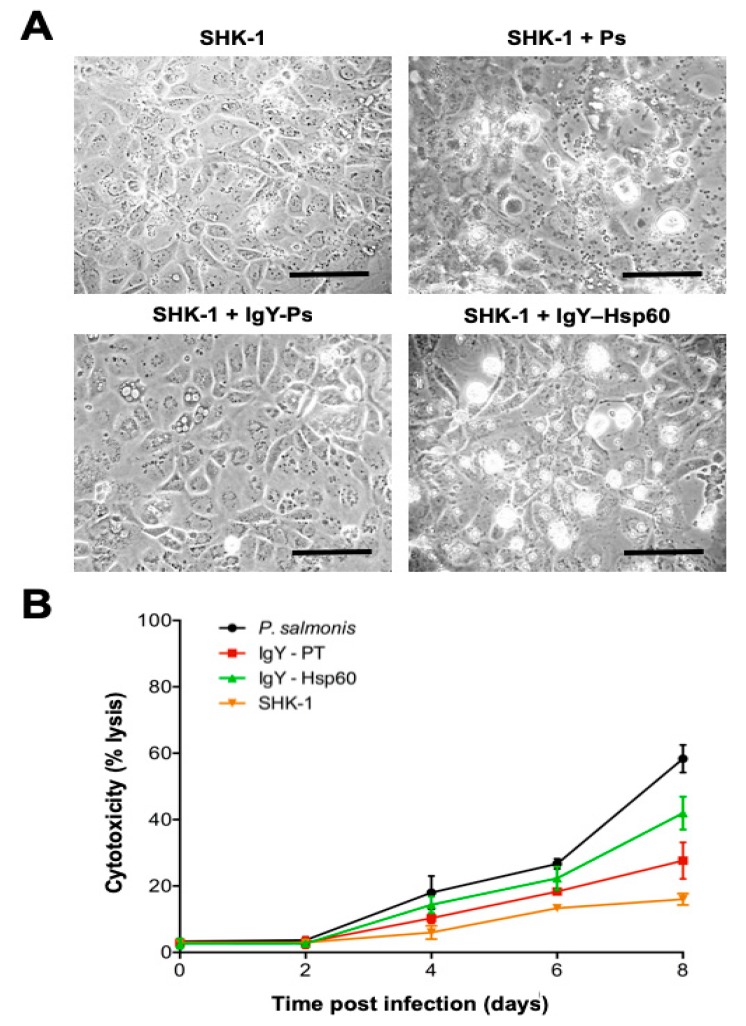
Analysis of protective effect generated by IgY anti-Hsp60 against *P. salmonis* infection. Assays were conducted for eight days in (**A**) non-infected SHK-1 cells (SHK-1), SHK-1 cells infected with *P. salmonis* (SHK-1 + Ps), SHK-1 cells pre-treated with IgY against *P. salmonis* total proteins (SHK-1 + IgY-Ps), or SHK-1 cells pre-treated with IgY against Hsp60 (SHK-1 + IgY-Hsp60). (**B**) The cytotoxicity of infected SHK-1 cells was measured by LDH release. Data are given as the mean ± standard deviation from three independent experiments. Bar scale = 50μm.

To evaluate the protective capacity of the IgY antibody against *P. salmonis* Hsp60, an in vitro inhibitory assay was performed using a synthetic peptide of Hsp60. The peptide was biologically active in inducing expression of the *il-1β* and *tnf-a* genes in salmon head kidney 1-derived (SHK-1) cells (Supplementary Figure S1). In SHK-1 cells treated with IgY against the Hsp60 peptide, the overall cytopathic effect observed was lower than that in untreated cells, but higher than the effect observed in cells pre-treated with IgY + total proteins (Figure 3A). In contrast, no protective effects were detected for infected SHK-1 cells treated with non-immune IgY or IgY against fructose-1,6-bisphosphatase 1 (data not shown). SHK-1 cells treated with IgY against total *P. salmonis* protein had an appearance similar to that of the uninfected controls. Supernatant lactate dehydrogenase (LDH) values for infected cells without IgY treatment increased after the second day of infection and reached ~60% cytotoxicity at eight days post-infection (Figure 3B). *P. salmonis*-infected cells treated with IgY against the whole protein extract of *P. salmonis* (IgY-PT) showed low cytotoxicity (18–30%), very similar to the levels observed in uninfected controls throughout the eight days post-infection (~20%). Importantly, cells treated with IgY against Hsp60 showed lower LDH values (between 35% and 45%) than that of the untreated positive control, but higher than those observed for the IgY-PT-treated cells.

## 4. Discussion

*P. salmonis* invades and replicates inside fish cells. HSP family members are major antigens in various bacterial pathogens that stimulate host immunogenic responses [40]. It is known that the subcellular location of Hsp60 is mainly cytoplasmic in eukaryotic and bacterial cells. However, for several intracellular pathogenic bacteria, Hsp60 is located in the periplasm, on the bacterial surface, and, occasionally, it can also be found as a secreted protein in the extracellular compartment during infection [14,41]. In this study, the immunogold analyses mainly located Hsp60 in the bacterial cytoplasmic space (~60%). Interestingly, ~28% of Hsp60-positive spots were identified in the cell envelope of *P. salmonis*. However, the low amount (~11%) of surface-localized Hsp60 could be due to bacterial cell lysis during growth, and not to export via secretion or other mechanisms.

On the other hand, increased Hsp60 expression occurs in bacterial membranes under stressful conditions, including *L. pneumophila* [42], *Borrelia bugdorferi* [41], and *Synechocystis* sp. [43], among others. Furthermore, Hsp60 exists on the surface of several bacterial pathogens, such as *H. pylori* [44] and *S. typhimurium* [45], thereby mediating the host-cell adhesion. Likewise, *L. pneumophila* entry to HeLa cells are mediated by Hsp60, which is highly secreted inside the host cytoplasmic vacuole [14]. Interestingly, secreted Hsp60 can interact with S-adenosylmethionine decarboxylase, an essential yeast enzyme required for polyamines synthesis in *Saccharomyces cerevisiae*, and necessary for the optimal intracellular growth of *L. pneumophila* [19].

Considering the increased expression and high antigenicity of *P. salmonis* Hsp60, our research sought to address the possibility of this protein being produced by an already known secretion system in *P. salmonis*. In this regard, a lack of leader sequences, secretion signal peptides, or other motifs have been reported, suggesting secretion of Hsp60 by *P. salmonis* [21]. Additionally, in silico predictions have been conducted for effector proteins of T3SS, T4SS, and T6SS, the most studied in Gram-negative pathogenic bacteria. It has been shown that this bacterium uses intracellular multiplication/defect in organelle trafficking (Icm/Dot) T4SS to translocate effectors that could modulate several host-cell functions [46]. The bioinformatics tools employed in this study consider several protein sequences features, including positional sequence patterns, charged residue biases, hydrophobicity, and motif conservation in C- and N-terminal residues, the primary focus of effector predictor tools [32,47,48,49]. Thus, the evidence indicates that Hsp60 would not be an effector translocated by the T4S, T3S, or T6S systems. Nonetheless, we have argued elsewhere [50] that secretion mechanisms for chaperonins should be naturally inefficient, to prevent depletion of an essential protein from the bacterial cytoplasm. However, the strong inflammatory immune response induced by a synthetic peptide of Hsp60, similar to that shown by other reports [20,51], the immune protection generated by a recombinant Hsp60-based vaccine against *P. salmonis* [22], and the decrease in the in vitro cytopathic effect conferred by IgY antibodies against an immunogenic peptide from *P. salmonis* Hsp60, all demonstrate the immunomodulatory activities of *P. salmonis* Hsp60 in host cells, and suggest that Hsp60 might be secreted by another *P. salmonis* mechanism. Thus, while *P. salmonis* seemingly does not secrete Hsp60, the presence of this protein in the bacterial cell envelope remains intriguing due to potential Hsp60 interactions with host cell proteins, possible mediator roles in host adhesion, and/or invasion, or modifying functions of specific proteins in the host cell. However, these possibilities were not assessed in the present study, and further experiments are needed to elucidate the putative function of surface-exposed Hsp60 during *P. salmonis* pathogenesis.

In turn, the production of biofilms in *P. salmonis* [52] has been reported to be a crucial mechanism for bacterial survival/pathogenesis [53,54]. Likewise, the relation between bacterial biofilm formation and OMVs is a well-recognized biofilm component found in several microorganisms, including *Francisella* spp. [55], *P. aeruginosa* [56], and *H. pylori* [57]. Indeed, *H. pylori* OMVs importantly participate in extracellular matrix formation in strain TK1402 biofilms [58,59]. Recently published descriptions and characterizations of *P. salmonis* OMVs [60] have shown that Hsp60 is one of the most abundant proteins in these vesicles [61]. Similar to *P. salmonis*, *H. somni* also produces biofilm, and the antibodies against *H. somni* Hsp60 inhibit biofilm formation [62]. Several proteins involved in iron uptake were identified in *P. salmonis* OMVs. These proteins could play a key role in bacterium survival and appropriate fitness. Therefore, it is feasible that *P. salmonis* Hsp60 may be involved in biofilm production/maintenance and/or be important for successful pathogen growth. However, further studies on Hsp60 presence and function in the *P. salmonis* envelope and vesicles are required to expand on these observations.

In conclusion, this study determined the subcellular location of *P. salmonis* Hsp60. Additionally, these results suggest that Hsp60 is not an effector protein of the *P. salmonis* secretion systems. While the specific role of Hsp60 in pathogenesis was not clearly elucidated, the presence and abundance of Hsp60 in *P. salmonis* OMVs indicate a possible important biological function in interacting with host proteins and/or modulating biofilm formation, thus favoring bacterial pathogenesis. However, further studies are needed to explain the abundance of Hsp60 in *P. salmonis* OMVs, and also to establish the specific role of Hsp60, especially regarding functions outside *P. salmonis*.

## Figures and Tables

**Table 1:** Distribution of Hsp60 epitopes in typical *P. salmonis* sections, as detected by immunoelectron microscopy with monoclonal rabbit immunoreagents.

Subcellular Location	Distribution of Epitopes ^(1)^ (%)
Cytoplasm	1.49 ± 1.1 (61.2)
Cell envelope	0.67 ± 0.73 (27.5)
Extracellular surface	0.28 ± 0.48 (11.3)
Total	2.43 ± 1.17 (100)

^(1)^ Mean of epitopes in a cell section.

**Table 2:** Hsp60 identified in different subcellular compartments of *P. salmonis* by liquid chromatography-mass spectrometry (LC-MS/MS).

Cell Compartment	Protein Name	Xcor ^(1)^	Sequence Coverage (%) ^(2)^	Matched Peptides
Outer membrane	60 kDa Chaperonin Hsp60	12,01	6,00	2
Periplasm	60 kDa Chaperonin Hsp60	19,30	9,00	3
Inner membrane	60 kDa Chaperonin Hsp60	6,23	7,00	2
Cytoplasm	60 kDa Chaperonin Hsp60	2329,85	51,00	41
Outer membrane vesicles	60 kDa Chaperonin Hsp60	286,72	48,53	14

^(1)^ The peptide cross-correlation score (Xcor) 2.5 was used to filter SEQUEST results to obtain positive identifications. ^(2)^ Coverage of protein sequence by the peptides used for identification.

## Supplementary Materials

The following are available online at https://www.mdpi.com/2076-2607/8/1/117/s1, Figure S1: Biological activity of *P. salmonis* Hsp60 peptide. Relative expressions of (A) il-1ß and (B) tnf-α in SHK-1 cells stimulated with the Hsp60 peptide (100 µM) in the presence or absence of the NF-kB inhibitor BAY 11-7082. GAPDH and ß-actin were used to normalize the mRNA levels of Hsp60. Values represent the mean ± standard deviation from three independent experiments. Asterisks (*) indicate significant differences (*p* < 0.05), Figure S2: Immunolocalization of Hsp60 in *P. salmonis* outer membrane vesicles (OMVs). Electron micrograph of *P. salmonis* LF-89^T^ showing Hsp60 labeling in a forming OMV (arrow). Bar scale = 0.5 μm, Table S1: Primers used in this study, Table S2: Hsp60 peptides identified in different subcellular compartments of *P. salmonis*, Table S3: Identity (top) and similarity (bottom) matrix for bacterial Hsp60 proteins.
