# Supplementary material for: Subcellular Location of Piscirickettsia salmonis Heat Shock Protein 60 (Hsp60) Chaperone by Using Immunogold Labeling and Proteomic Analysis
Source: Microorganisms. 2020 Jan 15;8(1):117. doi: 10.3390/microorganisms8010117 (PMC7023422; doi:10.3390/microorganisms8010117)
Supplement: Supplementary file 1 [file microorganisms-08-00117-s001.pdf]

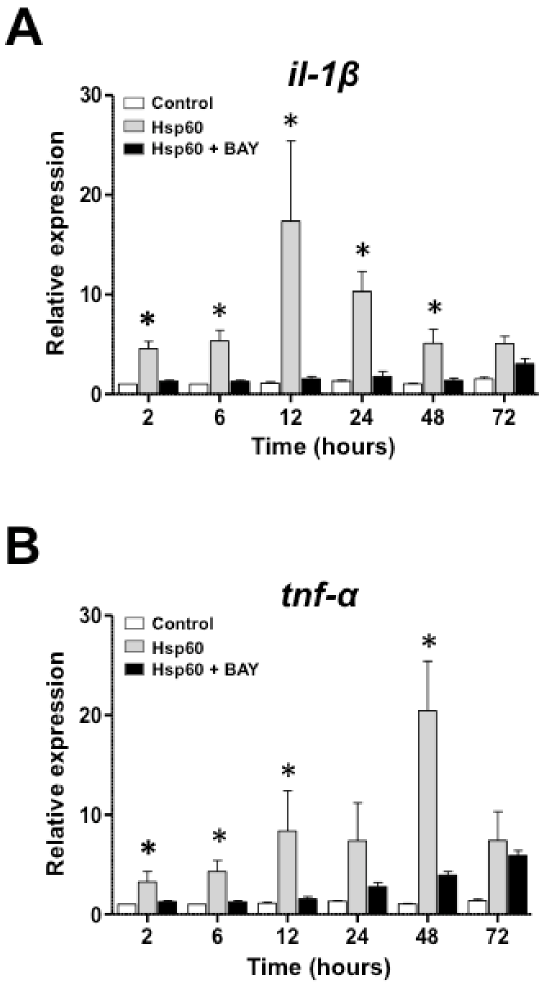

Suppl. Figure 1, Oliver et al

Supplementary Figure 1. Biological activity of *P. salmonis* Hsp60 peptide. Relative expressions of (A) il-1ß and (B) tnf-α in SHK-1 cells stimulated with the Hsp60 peptide (100 µM) in the presence or absence of the NF-kB inhibitor BAY 11-7082. GAPDH and ß-actin were used to normalize the mRNA levels of Hsp60. Values represent the mean ± standard deviation from three independent experiments. Asterisks (*) indicate significant differences ($p < 0.05$).

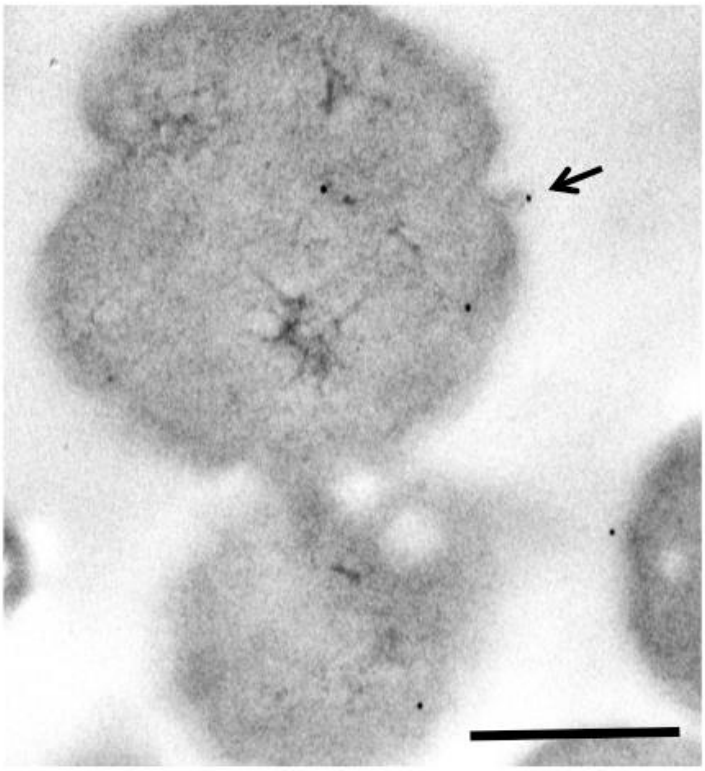

Suppl. Figure 2, Oliver et al

Supplementary Figure 2. Immunolocalization of Hsp60 in *P. salmonis* outer membrane vesicles (OMVs). Electron micrograph of *P. salmonis* LF-89^{T} showing Hsp60 labeling in a forming OMV (arrow). Bar scale = 0.5 μm.

Supplementary Table 1. Primers used in this study.

Primer	Sequence (5' – 3')	Gene
Il-1β F	CCA CCT GCT CAA CTT GC	*il-1β*
Il-1β R	GCA GCT CCA TAG CCT CAC TC	*il-1β*
TNF-α F	CGT GGT GTC AGC ATG GAA GA	*tnf-α*
TNF-α R	AGT ATC TCC AGT TGA GGC TCC ATT	*tnf-α*
23S F	TTG AAA ACC GGT GTT GAG AT	*23S*
23S R	CTC TAA CTG CCA AGG CAT CC	*23S*
β-actin F	GAC AAC GCA TCC GGT ATG TGC	*β-actin*
β-actin R	CAG CTC GTT GTA GAA GGT G	*β-actin*

Supplementary Table 2. Hsp60 peptides identified in different subcellular compartments of *P. salmonis*

Compartment	Protein	Xcor[(1)]	Sequence Coverage (%)[(2)]	Matched peptides	Peptides
Outer membrane	Hsp60/ Hsp60	12,01	6,00	2	KMLDGVNLLANAVKV
					KALEFANDEQAQGANILLRA
Periplasm	Hsp60/ Hsp60	19,30	9,00	3	KDNTTVIDGAGEQTAIEARV
					KALEFANDEQAQGANILLRA
					KMLDGVNLLANAVKV
Inner membrane	Hsp60/ Hsp60	6,23	7,00	2	KDNTTVIDGAGEQTAIEARV
					KALEFANDEQAQGANILLRA
Cytoplasm	Hsp60/ Hsp60	2329,85	51,00	41	AAVEEGVVPGGGVALVR
					ALDFANDEQAQGANILLR<br>AMAAVK<br>APGFGDRR<br>AQVEETSSDYDR<br>AQVEETSSDYDREK<br>ATIAAVAALK<br>ATLEHLGTAK<br>ATLEHLGTAKR

						DGVSVAK<br>DNTTVIDGAGEQTAIEAR<br>DRVDDALHATR<br>EIELSDK<br>EIELSDKFENMGAQMVK<br>ELLPTLESVAK<br>GIDKATIAAVAALK<br>GRNVILEK<br>GYLSPYFVNK<br>GYLSPYFVNKQEK<br>ISNIRELLPTLESVAK<br>KDRVDDALHATR<br>KISNIRELLPTLESVAK<br>LSGGVAVIK<br>MIAEIESPFILLVDK<br>MIAEIESPFILLVDKK<br>MLDGVNLLANAVK<br>MLDGVNLLANAVKVTLGPR<br>NVILEK<br>QIVENAGSEAAVILDK<br>QKMLDGVNLLANAVK<br>RGIDKATIAAVAALK<br>RIVVTK<br>SFGAPTITK<br>SFGAPTITKDGVSVAK<br>SVAAGMNPMDLK<br>SVAAGMNPMDLKR<br>VAKLSGGVAVIK<br>VDDALHATR<br>VGAATEIEMK<br>VGAATEIEMKEK<br>VTLGPR
Outer vesicles	membrane	Hsp60/ Hsp60	286,72	48,53	14	AIAQVGTISANSDEEIGSIIAK<br><br>SNDDAGDGTTTATVLAQAIIQEGVK<br>AIAQVGTISANSDEEIGSIIAK<br>DNTTVIDGAGEQTAIEAR<br>AQVEETSSDYDR<br>VSTDGVITVEEGSSLEnELDVVEGMqFDR<br>AMLEDIAILTGGTVISEEVGLnLEK<br>SGKPLFIIAEDVEGEALATLVVNNIR<br>EIELSDKFENMGAQMVK

					SVAAGMNPMDLK<br>DNTTVIDGAGEQTAIEAR<br>VGAATEIEMK<br>ATLEHLGTAK<br>SGKPLFIIAEDVEGEALATLVVNNIR

(1) The peptide cross correlation score (Xcor) 2.5 was used to filter SEQUEST results to obtain positive identifications.

(2) Coverage of protein sequence by the peptides used for identification.

Supplementary Table 3. Identity (top) and similarity (bottom) matrix for bacterial Hsp60 proteins

**Sequences**	**1**	**2**	**3**	**4**	**5**
1. WP_017377613.1 | [*P. salmonis* LF89]		74.0	73.6	62.2	56.4
2. KZX35693.1 | [*L. pneumophila*]	86.3		76.1	63.0	57.6
3. NP_820699.1 | [*C. burnetii* RSA 493]	86.1	87.7		61.6	59.2
4. ANH47662.1 | [*H. pylori*]	77.9	79.6	79.3		57.0
5. AIR13161.1 | [*M. tuberculosis* H37Rv]	72.9	74.1	75.5	73.4	
